# On the Treatment of Acne

**Published:** 1878-06

**Authors:** 


On the Treatment of Acne.—The following plan of treatment succeeds in a large number ofcases:—1] The face should be steamed every night by holding it over a basin of hot water for a few minutes. 2] The skin should then be well rubbed for five or ten minutes with soap and flannel, or a soft nail-brush may be used with advantage when the skin willbear it; the soap should then be sponged off with warm water. 3] When the face has been dried, a lotion, composed of half an ounce of precipitated sulphur, two drachms of glycerine, one ounce of spirits of wine, three ounces each of lime-water and rose-water, should be thoroughly applied and allowed to dry, and remain on all night. If the skin is greasy the addition of some ether to the lotion is an advantage. Sometimes an ointment is more effective than a lotion; in that case one drachm and a half of hypochloride of sulphur, ten grains of carbonate of potash, ten drops of oil of bitter almonds, and an ounce of lard may be used; or three drachms of sulphur ointment and five drachms of vaseline will be found to be a very useful unguent. Whatever is used should be allowed to remain on all night, and washed off in the morning with warm oatmeal and water or weak gruel. If the skin becomes very tender under this treatment, it may be discontinued for one or two nights and then resumed. The most common cause of failure is want of perseverance or timidity on the part of the patient or doctor, for a temporary increase in the redness and irritability of the skin often prevents the continuance of the most efficacious remedies.

In nearly all cases the local treatment of acne must for a long time be more or less continuous, inasmuch as when left to itself the disease is apt to return, but instead of using the remedies every night, it will be sufficient if they are applied once or twice a week. —*The Lancet, April* 1878.

